# Inhibiting the cGAS‐STING pathway in myeloid cells effectively improves myocardial healing related to TET2 deficiency‐induced DNA damage response

**DOI:** 10.1002/ctm2.1741

**Published:** 2024-06-22

**Authors:** Yaling Dou, Yan Zhang, Logan Rivera, Tingting Hong, Shaohai Fang, Thuy Tien Tran, Yubin Zhou, James F. Martin, Yun Huang

**Affiliations:** ^1^ Institute of Biosciences and Technology Texas A&M University Houston Texas USA; ^2^ Department of Integrative Physiology Baylor College of Medicine, Texas Heart Institute Houston Texas USA; ^3^ Department of Translational Medical Sciences School of Medicine Texas A&M University Houston Texas USA

**Keywords:** cGAS‐STING, epigenetics, myocardial repair, TET2

## Abstract

**Background:**

Myeloid cells play critical roles in the regulation of myocardial injury and repair. Clonal hematopoiesis (CH)‐related mutations in genes, such as Ten‐eleven Translocation 2 (TET2), can impair myeloid cells and are associated with increased risk of cardiovascular disease (CVD). How Tet2 loss‐of‐function (LOF) impacts myeloid cells and disrupts normal myocardial repair remains unclear.

**Methods:**

We established ischemia‐induced myocardial infarction (MI) in a myeloid‐specific Tet2‐deficient mouse model. The echocardiographic assessment was conducted to evaluate the cardiac function. Histological analysis was performed to evaluate morphological changes in infarcted areas and fibrosis. To monitor the dynamic changes of myeloid cells in cardiac tissues during cardiac remodeling after MI, we performed longitudinal analysis on subsets of myeloid cells using flow cytometry. We performed immunofluorescence (IF) staining to examine the DNA damage and genome instability caused by Tet2 LOF. Gene expression was assessed by real‐time qRT‐PCR. Stimulator of Interferon Genes (STING) pathway activation was assessed using various methods, including Western blotting, flow cytometry, ELISA and IF staining of key signaling proteins involved in this pathway. Additionally, H‐151 was used as a pharmacological tool to antagonize augmented STING activation in the murine MI model.

**Results:**

We observed a substantial increase of neutrophils in the post‐MI mice, which contributes to adverse outcomes during heart repair. Mechanistically, Tet2‐deficient myeloid cells exhibited increased genome instability, accompanied with augmented activation of the STING pathway. Furthermore, the use of H‐151 a covalent STING binder that targets the cysteine residue at position 91 and functions as a potent STING antagonist, led to a substantial decrease in neutrophil populations in Tet2‐deficient mice following myocardial infarction, thereby reversing adverse cardiac outcomes.

**Conclusion:**

Our novel findings establish the rationale for targeting the cGAS‐STING pathway as a promising therapeutic strategy to mitigate cardiovascular disease risk in individuals with clonal hematopoiesis harboring TET2 loss‐of‐function mutations.

**Highlights:**

1. Myeloid‐specific Tet2 depletion promotes neutrophil expansion upon myocardium infarction (MI);
2. Tet2‐deficient myeloid cells exhibit increased genome instability and cGAS‐STING overactivation;
3. STING antagonist H‐151 treatment reduces neutrophil expansion in Tet2‐deficient mice after MI and mitigates deleterious cardiac outcomes.

## Graphical abstract

In this study, we analysed the dynamics of myeloid cells in a myeloid‐specific Tet2‐deficient mouse model after ischaemia‐induced myocardial infarction (MI). We observed a substantial increase of neutrophils in the post‐MI mice, which contributes to adverse outcomes during heart repair. Mechanistically, Tet2‐deficient myeloid cells exhibited increased genome instability, accompanied with augmented activation of the cGAS‐STING pathway. Inhibition of STING resulted in significantly reduced neutrophils in Tet2‐deficient mice after MI to enable reversal of deleterious cardiac outcomes. Therefore, targeting the cGAS‐STING pathway is a viable therapeutic option to reduce the risk of cardiovascular diseases in CH individuals bearing TET2 LOF mutations.

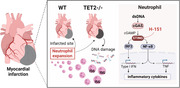

## 1 INTRODUCTION

Cardiovascular disease (CVD) is a leading cause of death globally.^1^, ^2^, ^3^ Atherosclerosis and myocardial infarction (MI) represent two major CVDs with high mortality rates, which are considered as not merely lipid or metabolic disorders but are also correlated with the dysregulation of the hematopoietic and immune systems.^4^, ^5^ Indeed, recent studies have shown that aged individuals with clonal haematopoiesis (CH) show increased risk of developing atherosclerotic CVDs.^6^, ^7^ In vivo studies conducted in transgenic mice have further confirmed that dysfunction of myeloid cells, such as macrophages as seen in CH of indeterminate potential carriers, is associated with a high risk of CVD.^8^, ^9^ Myeloid cells are intimately implicated in myocardial repair. Different subsets of myeloid cells, such as neutrophils, macrophages and monocytes, play distinct roles during this reparative process.^10^, ^11^ For instance, neutrophils have been shown to act as the first responders to MI‐mediated cardiac tissue damage by massively infiltrating into the infarcted area to orchestrate macrophages towards a reparative phenotype.^12^, ^13^ On the flip side, excessive neutrophil infiltration is associated with adverse clinical outcomes and high mortality in patients experiencing MI.^14^ The homeostasis and collaborative interactions of myeloid cells in the infarcted area are critical for tissue repair in response to acute myocardial injury.

Recent studies reported that CH has emerged as a major independent risk factor in CVD and heart failure.^6^, ^7^ CH‐associated somatic mutations in hematopoietic stem progenitor cell (HSPC) alter the function of its progenies, such as myeloid cells, and subsequently promote inflammasome activation in peripheral organs, including the cardiovascular system. In CH individuals, Ten‐Eleven Translocation 2 (TET2) is among the most frequently mutated genes in HSPCs and is associated with CVD pathogenesis.^7^ TET2 belongs to the methylcytosine dioxygenase family that catalyses the oxidation of 5‐methylcytosine in the mammalian genome to ultimately cause DNA demethylation.^15^, ^16^, ^17^ Tet2 deletion has been shown to cause the upregulation of pro‐inflammatory IL‐1β and augment NOD‐, LRR‐ and pyrin domain‐containing protein 3 (NLRP3) inflammasome activation in myeloid cells to accelerate heart failure.^9^ Furthermore, Tet2 deficiency has been found to enhance type I interferon response during MI,^18^ but the underlying molecular mechanism remains not fully resolved. More specifically, how Tet2 loss in myeloid cells causes abnormalities in innate immune response during MI is yet to be clarified.

In this study, we systematically examined the impact of Tet2 deletion on subsets of myeloid cells during MI. We discovered that the neutrophil is one of the most significantly affected cell populations upon Tet2 depletion during the acute phase post‐MI, which might account for worse outcomes in mouse models of MI with myeloid‐specific Tet2 ablation. At the molecular level, we observed notable impaired genome stability and increased DNA double‐stranded breaks (DSBs) in Tet2‐deficient myeloid cells at infarcted areas, which triggered the activation of the cyclic guanosine monophosphate‐adenosine monophosphate (GMP‐AMP) synthase (cGAS)‐stimulator of interferon gene (STING) pathway. More importantly, antagonizing cGAS‐STING activation in myeloid‐specific Tet2 deficient mouse models led to prominent beneficial effects in response to MI. Collectively, our study provides a comprehensive and unbiased molecular portrait on different subsets of Tet2‐deficient myeloid cells within the infarcted areas following the acute induction of MI. We also unveiled a previously unrecognized mechanism by which Tet2 loss triggers innate immune response in myeloid cells to exacerbate cardiac injury. From a translational perspective, our findings point to an underexplored therapeutic strategy by targeting the cGAS‐STING pathway in individuals with CH‐associated heart disease.

## 2 RESULTS

### 2.1 Myeloid‐specific Tet2 depletion promotes neutrophil expansion upon MI induction

**FIGURE 1:**
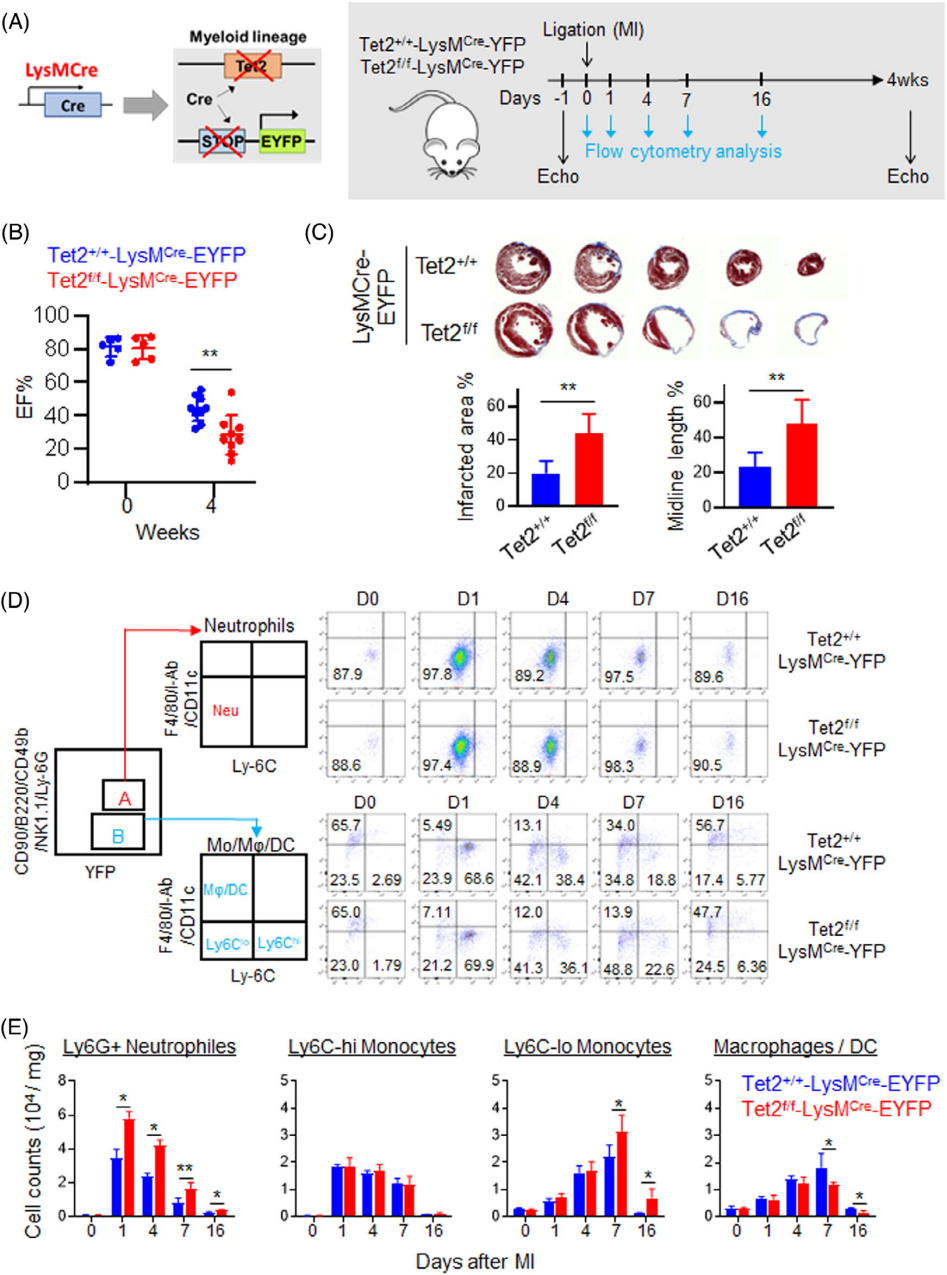
Myeloid‐specific Ten‐Eleven Translocation 2 (Tet2) deletion promotes neutrophil expansion following left anterior descending (LAD) ligation‐induced acute myocardial infarction (MI). Data were shown as mean  ±  SD (*p < 0.05; **p < 0.005; two‐sided unpaired Student's *t*‐test). (A) Schematic of the experimental design. (Left) Generation of a myeloid‐specific Tet2‐deficient mouse model with the co‐expression of enhanced yellow fluorescent protein (EYFP) as the indicator. (Right) The experimental timeline. (B) Quantification of the percentage of ejection fraction (EF%) for the control (LysMCre‐EYFP) and conditional Tet2‐KO (Tet2^f/f^‐LysMCre‐EYFP) groups at 0 or 4 weeks post‐myocardial infarction (MI) (*n* = 5−9). (C) Representative histological images (top) and statistical analysis results (bottom) for the indicated groups. Heart tissues and infarcted areas were evaluated at 4 weeks after MI (*n* = 5). (D) Flow cytometry analysis on subsets of myeloid cells, including neutrophils, monocytes (Ly6C‐high and Ly6C‐low) and macrophages/dendritic cells (DCs), in infarct areas. (E) Quantification of the population of Ly6G+ neutrophils, monocytes (Ly6C‐high and Ly6C‐low) and macrophages/DCs in infarct areas at 0‐, 1‐, 4‐, 7‐ or 16‐day post‐MI (*n* = 3–4).

To evaluate how Tet2 regulates the dynamics of myeloid cells in injured myocardium, we induced MI in LysMCre mice and LysMCre‐Tet2^f/f^ mice by permanent left anterior descending artery (LAD) ligation. To facilitat sensitive monitoring of myeloid cells in cardiac tissues, we introduced an enhanced yellow fluorescent protein (EYFP) gene floxed by a STOP sequence at the Gt(ROSA)26Sor locus into these transgenic mice (Tet2^+/+^‐LysMCre‐EYFP as control or Tet2^f/f^‐LysMCre‐EYFP [abbreviated as conditional Tet2‐KO]) (Figure 1A, Figure S1A). Consistent with a previous report,^9^ Tet2 depletion in the myeloid lineage led to worse cardiac remodelling/repair with significantly reduced ejection fraction (EF) 4 weeks after LAD ligation‐induced MI (Figure 1B and Table S1). Histological analysis further revealed that hearts obtained from the Tet2^f/f^‐LysMCre‐EYFP mice had larger infarcted areas and more fibrotic tissues (Figure 1C). To monitor the dynamic changes of myeloid cells in heart tissues during their response to cardiac remodelling after MI, we performed longitudinal analysis on subsets of myeloid cells in heart tissues using flow cytometry at Days 0, 1, 4, 7 and 16 post‐MI in LysMCre mice and LysMCre‐Tet2^f/f^ mice with the R26‐stop‐EYFP cassette (Figure 1A). We first carried out flow cytometry analysis on myeloid cells using a well‐documented labelling strategy via CD11b gating^10^ or gating on EYFP^+^ cells with Cre‐expression in the transgenic mice (Figure S1B). Both analysis strategies yielded similar results with clear separation of neutrophils, monocytes (Ly6C‐high and Ly6C‐low) and macrophages/dendritic cells (DCs) (Figure S1B). Given that EYFP gating enriched Cre‐expressing cells with Tet2 deletion, we moved on to gate EYFP^+^ cells to monitor the dynamic changes in the composition of Tet2‐deficient myeloid cells in heart tissues following MI (Figure 1D).

We observed a significant increase of neutrophils in heart tissues at post‐MI Day 1, followed by a gradual drop during post‐MI Days 4–16 (Figure 1E). Interestingly, myeloid‐specific Tet2 deletion resulted in approximately 1.5‐fold increase in neutrophil accumulation in injured myocardium at post‐MI Day 1 when compared to the control group (Figure 1E, Figure S1C). Compared to the control group, more appreciable neutrophil accumulation was noted in the conditional Tet2‐KO group, which persisted throughout the entire observation window (post‐MI Days 4–16; Figure 1E). In parallel, monocytes and macrophages/DCs experienced notable expansion at Days 1 and 4 post‐MI, but no significant difference was noted between the control and conditional Tet2‐KO groups. At Days 7 and 16 post‐MI, we observed a reduction in the macrophages/DCs population with accompanying increase in Ly6C‐low monocytes from the conditional Tet2‐KO heart tissues. This is likely due to the expansion of neutrophils in the cardiac tissues of Tet2^f/f^‐LysMCre mice at the acute phase post‐MI, which subsequently leads to enhanced and persistent inflammatory response at the infarct zone. Given that MI might also promote myelopoietic response in noncardiac tissues,^18^ we further evaluated subsets of myeloid cells in both bone marrow and peripheral blood collected from the same two groups of mice at the same time points. Similar to the phenotypes seen within the infarcted areas, we detected more expansion of neutrophils in both peripheral blood (PB) and bone marrow isolated from Tet2^f/f^‐LysMCre mice at Day 1 post‐MI (Figure S1D,E). Compared to the control, the conditional Tet2‐KO group exhibited a more prominent neutrophil expansion in the peripheral blood at Days 4 and 6 after MI (Figure S1D), indicating stronger MI‐induced activation of systematic innate immune response in these transgenic mice. Collectively, these findings indicate that myeloid‐specific Tet2 deletion impairs cardiac repair following LAD‐induced myocardial injury and leads to neutrophil expansion at infarcted zones and in noncardiac tissues during the acute phase after MI.

### 2.2 Tet2‐deficient myeloid cells had increased DNA damage

**FIGURE 2:**
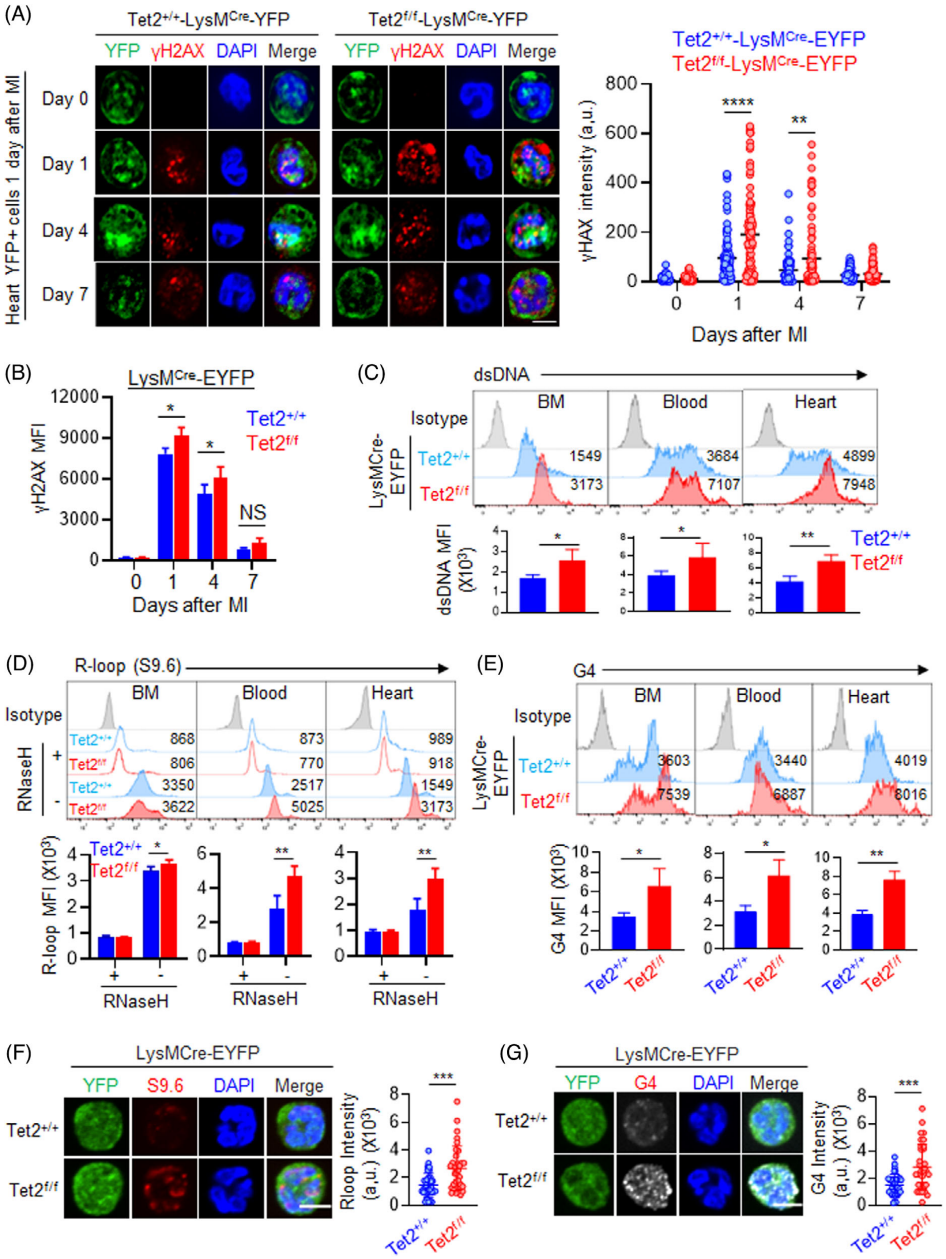
Ten‐Eleven Translocation 2 (Tet2)‐deficient myeloid cells exhibit increased DNA damage. (A) Representative immunofluorescence images (left) of EYFP^+^ cells purified from the control (Tet2^+/+^) and Tet2KO (Tet2^f/f^) infarcted heart tissues at Days 0, 1, 4, 7 and 16 following myocardial infarction (MI) procedures. (Right) Quantification of γH2AX levels in the indicated groups (*n* = 100 cells). Scale bar: 5 μm. Data were shown as mean  ±  SD (****p < 0.0001, **p < 0.005; two‐sided unpaired Student's *t*‐test). (B) Quantification of the mean fluorescence intensity (MFI) of γH2AX levels for the indicated groups (*n* = 3). Data were shown as mean  ±  SD (*p < .05; two‐sided unpaired Student's *t*‐test). (C) Quantification of the double‐stranded DNA (dsDNA) levels in EYFP^+^ cells obtained from the bone marrow (BM), peripheral blood and infarcted areas at Day 1 post‐MI. (Top) Representative flow cytometry profiles. (Bottom) Statistical analysis results (*n* = 4). Data were shown as mean  ±  SD (**p < 0.005, *p < 0.05; two‐sided unpaired Student's *t*‐test). (D and E) Flow cytometry analysis on the R‐loop (D) and G‐quadruplex (G4) (E) levels in EYFP^+^ cells obtained from bone marrow (BM), peripheral blood and infarcted areas of the indicated groups. (Top) Representative flow cytometry profiles. (Bottom) Statistical analysis results (*n* = 3). Data were shown as mean  ±  SD (**p < 0.005, *p < 0.05; two‐sided unpaired Student's *t*‐test). (F and G) Immunofluorescence staining of R‐loop (F) and G‐quadruplex (G4) (G) levels in EYFP^+^ cells from infarcted areas of the control and Tet2KO mice at Day 1 after MI. (Left) Representative flow cytometry panels. (Right) Statistical analysis results (*n* = 35 cells). Scale bar, 5 μm. Data were shown as mean  ±  SD (***p < 0.0005; two‐sided unpaired Student's *t*‐test).

As Tet‐deficient cells tend to exhibit increased genome instability and DNA damage,^19^, ^20^, ^21^ we performed immunofluorescence staining of phosphorylated histone 2A.X (γH2AX), a marker used for monitoring DSBs during DNA damage,^22^ in EYFP^+^ cells purified from injured myocardium before and after MI induction for 1, 4 and 7 days. We observed dynamic changes of the γH2AX level in EYFP^+^ myeloid cells purified from injured sites in the control mice (Figure 2A). γH2AX staining showed immediate increase in EYFP^+^ myeloid cells isolated from injured sites at Day 1 post‐MI, followed by a gradual reduction from Days 4 to 7. A similar scenario was visualized in the conditional Tet2‐KO group, but the immunostaining intensities at both Days 4 and 7 were significantly stronger than the control group (Figure 2A). This finding was independently validated by flow cytometry analysis via quantitative measurements of the intracellular γH2AX levels in EYFP^+^ myeloid cells purified from infarct areas of both the control and Tet2‐deficient mice before and after MI at the same time points (Figure 2B). In parallel, we measured active caspase‐3 and Annexin V/7‐AAD staining intensities with flow cytometry in control and Tet2‐deficient cells isolated from the infarcted area at Day 1 after MI (Figure S2A). No significant difference was observed between the control and Tet2‐deficient groups, suggesting that the increased γH2AX level is not likely due to cell death or apoptosis.

Because sterile tissue injury could also remotely trigger innate immune response in bone marrow and peripheral organs,^18^ we next extended our analysis to EYFP^+^ cells isolated from bone marrow and peripheral blood before and after MI induction (Figure S2B). Indeed, we observed similar dynamic changes of the γH2AX level in EYFP^+^ myeloid cells from these tissues during post‐MI cardiac repair. Again, myeloid‐specific Tet2 deletion led to increased γH2AX levels in both bone marrow and peripheral blood at Day 1 post‐MI (Figure S2B,C). We next sought to confirm increased DNA damage by directly measuring the double‐stranded DNA (dsDNA) levels in EYFP^+^ cells purified from infarcted areas, bone marrow and peripheral blood at Day 1 post‐MI. Similar to the previous use of γH2AX as readout, we detected a more prominent increase in the dsDNA level in the conditional Tet2‐KO group than in the control group (Figure 2C, Figure S2D). To further confirm altered genome stability arising from Tet2 deletion, we compared the levels of R‐loops and G‐quadruplexes (G4s), two types of nucleic acid structures indicative of genome instability,^23^, ^24^ in the control and Tet2‐deficient EYFP^+^ myeloid cells after MI. When compared to the control group, more pronounced accumulation of R‐loops and G4 was detected in Tet2‐deficient EYFP^+^ myeloid cells derived from bone marrow, peripheral blood, or infarct areas at Day 1 after MI (Figure 2D–G). As the antibody (S9.6) recognizing R‐loop has been reported to cross‐react with single‐stranded RNA,^25^ we ruled out this possibility by treating cells with RNaseH, which is known to specifically recognize R‐loop and subsequently remove DNA:RNA hybrids.^26^ We observed notable loss of R‐loop signals after RNaseH treatment in both the control and Tet2‐deficient groups, confirming that the S9.6 antibody immunostaining signals could reflect R‐loop dynamic changes (Figure 2D). Together, our findings establish that Tet2 deficiency could severely impair DNA damage repair and promote genome instability in myeloid cells in response to myocardial infarction.

### 2.3 Tet2 deficiency leads to augmented activation of the cGAS‐STING pathway

**FIGURE 3:**
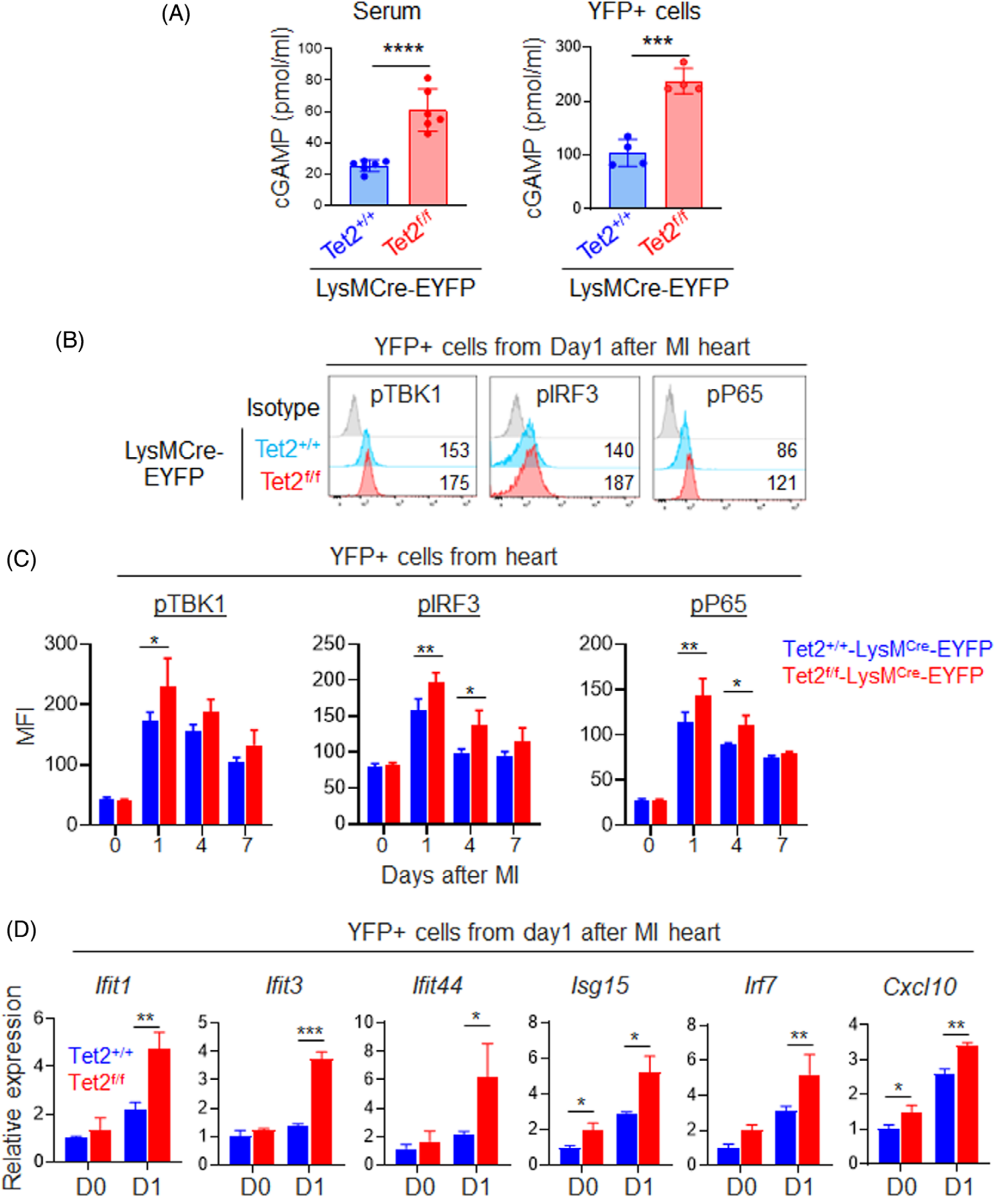
Ten‐Eleven Translocation 2 (Tet2) deficiency leads to augmented activation of the GMP‐AMP synthase (cGAS)‐stimulator of interferon gene (STING) pathway. Data were shown as mean  ±  SD (****p < 0.0001, ***p < 0.0005, **p < 0.005, *p < .05; two‐sided unpaired Student's *t*‐test). (A) The quantification of cyclic GMP‐AMP (cGAMP) levels measured by ELISA in sera and EYFP^+^ cells obtained from the control and conditional Tet2‐KO mice at Day 1 after myocardial infarction (MI) (*n* = 3–6). (B) Flow cytometry analysis on the levels of phosphorylated TBK1 (p‐TKB1), p‐IRF3 and p‐P65 in EFYP+ cells isolated from the control and Tet2‐KO mice at Day 1 after MI. (C) The statistical quantification of the mean fluorescence intensity (MFI) measured by flow cytometry, indicating the levels of phosphorylated TBK1, IRF3 and p65 in EFYP+ cells purified from the infarcted areas at Days 0, 1, 4 and 7 after MI (*n* = 3). (D) The quantitative real‐time PCR analysis on the indicated genes in EFYP+ cells purified from control and Tet2KO mice before and at Day 1 after MI (*n* = 3).

It is established that intracellular dsDNA can induce the innate immune defence programme by activating the cyclic cGAS – STING pathway.^27^ We reasoned that elevated dsDNA due to augmented genome instability might lead to cGAS‐STING activation in Tet2 deficient myeloid cells to exacerbate inflammation. As the activation of the cGAS‐STING pathway is initiated by the binding of dsDNA to cGAS with subsequent production of 2′3′ cyclic GMP‐AMP (cGAMP),^28^, ^29^ we first assessed cGAMP production in EYFP^+^ cells isolated from infarct areas at Day 1 post‐MI. Compared to the control group, we detected elevated levels of cGAMP in EYFP^+^ cells purified from infarct areas, as well as in the serum, at Day 1 post‐MI in the Tet2‐deficient groups (Figure 3A). Because the expression level of intracellular cGAS (Figure S3A) and its subcellular localization (cytosol‐to‐nucleus ratio; Figure S3B) were not altered upon Tet2 depletion, we ruled out the possibility that the increased production of cGAMP arises from differential expression or abnormal subcellular distribution of the cGAS protein per se.^30^

To further examine the activation status of the downstream effectors in the cGAS‐STING pathway, we monitored the phosphorylation levels of TBK1 (p‐TBK1), IRF3 (p‐IRF3) and P65 (p‐P65) in EYFP^+^ cells purified at Days 0, 1, 4 and 7 post‐MI from infarct areas. We observed upregulation of phosphorylated TBK1, IRF3 and P65 in the Tet2‐deficient group compared to the control at Days 1 and 4 after MI (Figure 3B,C and Figure S3C). The similar upregulation in phosphorylated IRF3 and P65 was also observed in EYFP^+^ cells purified from the peripheral blood in Tet2‐deficient mice at Day 1 post‐MI (Figure S3D), supporting the conclusion that MI‐induced systematic activation of the cGAS‐STING pathway in myeloid cells. The most prominent downstream event following cGAS‐STING activation is the transcriptional activation of type I interferon and interferon‐stimulated genes (ISGs).^31^ We evaluated the transcriptional expression levels of ISGs (*Ifit1*, *Ifit3*, *Ift44*, *Isg15*, *Irf7* and *Cxcl10*) in EYFP^+^ cells obtained from infarct areas at Days 0 and 1 after MI. Indeed, we observed strong upregulation of these genes in the Tet2‐deficient mice compared with the control group at Day 1 after MI (Figure 3D). In parallel, we measured the expression level of *Il6* and *Il1β*, which encode cytokines that are reported to be upregulated in Tet2‐deficient myeloid cells,^9^, ^32^ in control and Tet2‐deficient EYFP^+^ cells at Day 1 after MI (Figure S3E). We did not observe significant transcriptional alterations in *Il6* and *Il1β* between the control and Tet2‐deficent conditions, possibly because of the early stage of MI. Overall, our data strongly suggests that elevated dsDNA production due to increased genome instability in Tet2‐deficient myeloid cells readily activated the cGAS‐STING pathway after MI.

### 2.4 H‐151 as a STING inhibitor curtails neutrophil expansion and improves post‐MI cardiac repair

As we observed strong cGAS‐STING activation in myeloid cells after MI, we hypothesized that suppressing the cGAS‐STING pathway would promote better myocardial repair following acute MI. To test this idea, we antagonized STING activation by using a compound, H‐151, which targets the ligand binding domain of STING to potently block cGAMP‐induced STING dimerization and downstream signaling.^33^

**FIGURE 4:**
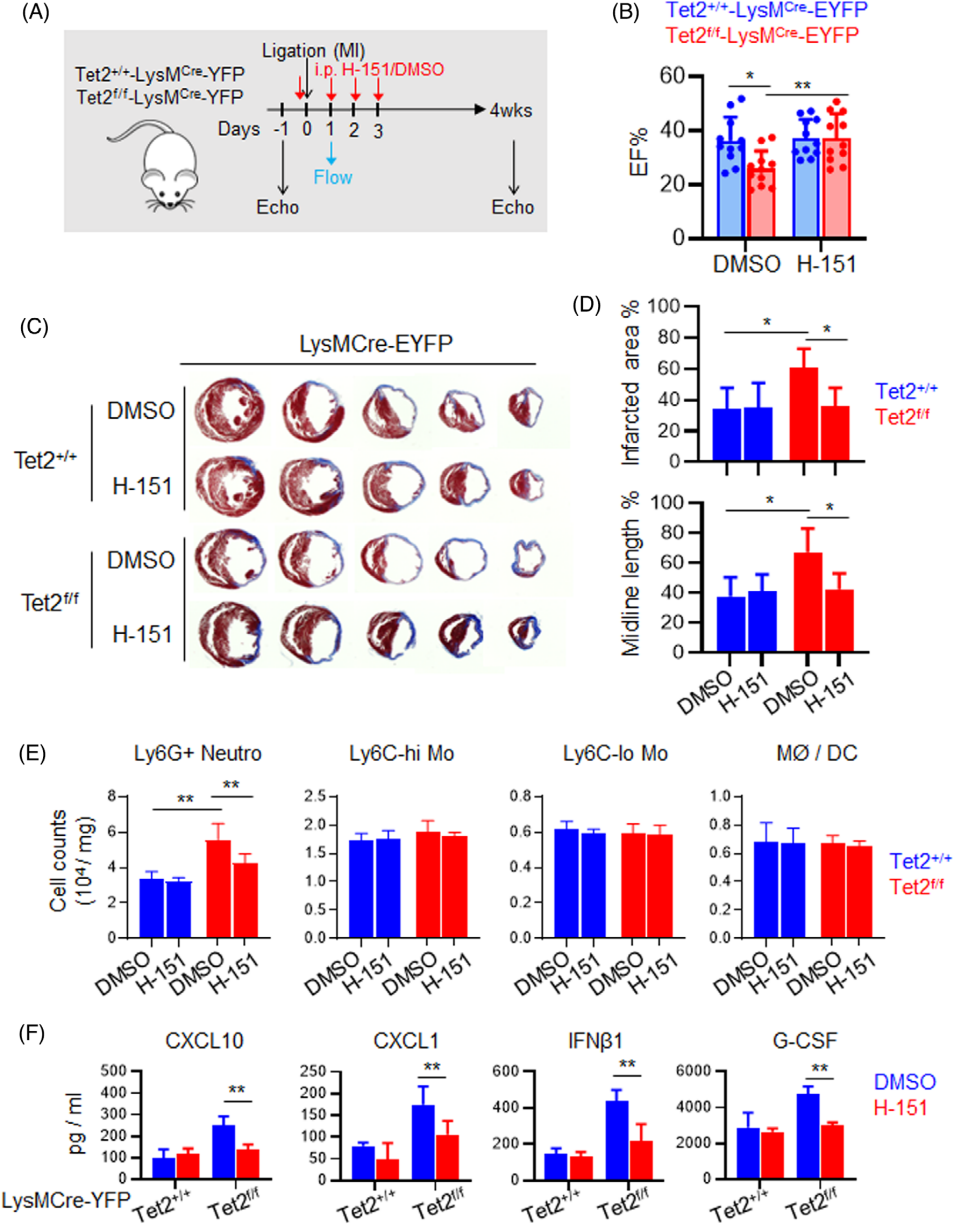
Antagonizing the stimulator of interferon gene (STING) pathway suppresses neutrophil expansion and improves cardiac remodelling in the myeloid‐specific Ten‐Eleven Translocation 2 (Tet2)‐deficient mouse model following acute myocardial infarction (MI). Data were shown as mean  ±  SD (**p < 0.005, *p < 0.05; two‐sided unpaired Student's *t*‐test). (A) Experimental design. H‐151 as a specific STING inhibitor or DMSO (as solvent control) was administered as indicated, followed by echocardiography imaging and histological analysis 4 weeks after MI. (B) Quantification of the ejection fraction (EF%) in the control (Tet2^+/+^‐LysMCre‐EYFP) and Tet2KO (Tet2^f/f^‐LysMCre‐EYFP) mice treated with DMSO or H‐151 at 4 weeks post‐MI (*n* = 11). (C and D) Representative histological images (C) and statistical analysis (D) on heart tissues and damaged sites collected from the control and Tet2‐KO mice 4 weeks after MI. The mice were treated with either DMSO or H‐151 (*n* = 5). (E) Quantification of the population of Ly6G+ neutrophils, monocytes (Ly6C‐high and Ly6C‐low) and macrophages / dendritic cells (DCs) in myocardial infarct areas of the control or Tet2KO mice treated with DMSO or H‐151 at Day 1 after MI (*n* = 3). (F) Quantification of the serum levels of the indicated cytokines in the control and conditional Tet2‐KO mice treated with DMSO or H‐151 on Day 1 after MI (*n* = 4).

We treated the control and myeloid‐specific Tet2‐deficient mice with H‐151 or DMSO (as solvent control) via daily intraperitoneal injection at acute phase of myocardial injury for four consecutive days and then monitored the myocardial repair 4 weeks after MI (Figure 4A). In Tet2‐deficient mice, H‐151 led to an increased EF (Figure 4B and Table S2), as well as significant reduction in the scar size and infarct area and the midline length compared with the DMSO group (Figure 4C,D), thus suggesting a strong cardio‐protective effect via suppression of STING activation.

As our initial analysis demonstrated that myeloid‐specific Tet2 deficiency primarily promoted the neutrophil expansion in the acute phase of myocardial injury, we compared the neutrophil levels within the infarct area at post‐MI Day 1 between the control and conditional Tet2‐KO groups following treatment. We found that H‐151 treatment significantly reduced neutrophil expansion in the infarct area but exerted minor effects on other types of myeloid cells involved in ventricular healing at later stages after MI, including monocytes and macrophages/DCs (Figure 4E). Furthermore, H‐151 treatment caused a reduction in the serum levels of cytokines, such as Cxcl10, Cxcl1, IFNβ1 and G‐CSF, in the Tet2‐deficient group (Figure 4F). Overall, these data point to a promising therapeutic potential of targeting the cGAS‐STING pathways at acute phase during myocardial injury to benefit ventricular healing in the genetic background of myeloid‐specific Tet2 deficiency.

## 3 DISCUSSIONS

The association between CH and the risk of developing CVD has been reported previously.^6^, ^7^ CH‐associated TET2 loss‐of‐function (LOF) has been shown to accelerate heart failure and atherosclerosis development in mouse models by impairing macrophage function, pointing to the possibility of targeting CH‐associated mutant innate immune cells to mitigate CVD risk.^8^, ^9^ Due to the complexity of the innate immune response post‐MI, identification and characterization of the subsets of myeloid cells that are directly affected by TET2 LOF have not been reported. Such an insight would fill a critical knowledge gap in CH/CVD pathogenesis and provide alternative approaches for therapeutic intervention of CH. To achieve this, we generated a LysMCre‐Tet2f/f mouse model in the current study to investigate the direct impact of myeloid‐specific Tet2 LOF after MI. By systematically analysing dynamic changes of subsets of Tet2‐deficient myeloid cells in response to MI, we identified an expansion of neutrophils in Tet2‐deficient myeloid cells after MI not only in infarct areas but also in peripheral blood and bone marrow. Our study reveals the neutrophil as one of the major cell populations in the myeloid lineage that is directly impacted by Tet2 loss, which might account for maladaptive cardiac remodelling observed in the mouse model of acute MI.

The roles of neutrophils in response to MI remain inadequately understood.^13^ Massive expansion and infiltration of neutrophils into the infarct area occur within the first few hours and peak at 24 h after the onset of cardiac ischaemia. Neutrophils can generate high levels of reactive oxygen species and proteolytic enzymes to aggravate the local tissue damage.^34^ Meanwhile, infiltrated neutrophils at infarct area coordinate the monocyte and macrophage plasticity during both inflammatory and reparative processes.^12^ Depletion of neutrophils might lead to unresolved inflammation and impair scar formation during cardiac tissue repair.^12^ In the present study, we observed massive expansion of neutrophils not only in infarct areas but also in peripheral blood and bone marrow in myeloid‐specific Tet2‐deficient mice following acute MI, suggesting a systematic inflammation triggered by cardiac tissue damage. In the same mouse model, we also observed expansion of Ly6C‐low monocytes only in the infarct area, but not in the peripheral blood and bone marrow. This might be due to the massive infiltration of neutrophils at earlier time points after LAD ligation in Tet2‐deficient mice, which triggers severe local tissue damage and requires more Ly6C‐low monocytes to attenuate neutrophil‐mediated inflammatory response at infarct area. These data support the idea that neutrophils influence monocyte recruitment to the infarct area in response to cardiac tissue damage. Fine‐tuning the neutrophil level and activity might be pursued as a potential therapeutic strategy, in which one would need to find a balance between inflammatory and reparative responses following MI. It is important to acknowledge that although this study focuses on investigating myeloid‐specific deletion of Tet2 and its impact on neutrophil expansion during the early stages of MI, Tet2 might play additional roles in regulating other myeloid cells, such as macrophages and DCs, in response to tissue damage‐induced innate immune response. Further investigations are warranted to delve deeper into these aspects.

Two recent studies suggested the promising therapeutic potential of targeting NLRP3/IL‐1β inflammasome in TET2 LOF‐mediated CH‐associated CVD, including atherosclerosis and heart failure.^8^, ^9^ In addition to NLRP3 inflammasome‐mediated innate immune response to sterile tissue damage, other signalling pathways might be actively involved in initiating the innate immune and inflammatory cascade in response to stress‐induced damage.^35^ In this study, we unveiled that the cGAS‐STING pathway could also be explored as a potential therapeutic target in order to ameliorate MI‐induced tissue damage in the context of Tet2 LOF. Although our study suggests that Tet2 LOF‐mediated genome instability induces cGAS‐STING activation, it is important to note that other non‐mutually exclusive mechanisms may further contribute to the observed abnormalities, including, but not limited to, Tet2 LOF‐induced epigenetic changes and transcriptional alterations of genes involved in innate immunity. Further follow‐on multi‐omic studies might provide insights into the epigenetic and transcriptional changes occurring in Tet2‐deficient myeloid cells in response to MI.

At the molecular level, we observed augmented activation of cGAS‐STING signalling in Tet2‐deficient myeloid cells, owing to Tet2 deficiency‐induced enhancement of genome instability and increased intracellular dsDNA after infarction. Furthermore, previous studies have reported the activation of cGAS‐STING in myeloid cells in response to tissue damage, as observed in animals experiencing MI.^36^ Although the protective effect of targeting the cGAS‐STING pathway during myocardial ischaemic damage and repair has been reported previously,^36^ out data further suggest that suppressing STING activation could further benefit the myocardial repair in the context of Tet2 dysfunction in myeloid cells, as found in CH individuals with TET LOF‐mutations. Both the NLRP3/IL‐1β inflammasome and cGAS‐STING signalling contribute to innate immunity. Previous studies suggested that these pathways can be mutually regulated. For example, IL‐1β production has been reported to activate cGAS‐STING signalling in human epithelial and myeloid cells,^37^ whereas IL‐1β gene expression is also regulated by STING‐dependent signalling in colon tissues.^38^ Furthermore, suppressing either IL‐1β or STING could benefit the treatment of chronic kidney diseases.^39^ Future studies are needed to sort out how NLRP3/IL‐1β inflammasome and/or the cGAS‐STING pathway contribute to innate immune response and cardiac remodelling, thereby guiding more rational design of better therapeutics to combat cardiac ischaemic damage.

Our study primarily focuses on the immediate injury response following MI, particularly emphasizing the impact of Tet2 loss on myeloid cells during the acute phase. Although our findings provide insights into the neutrophil‐driven inflammation and cGAS‐STING activation post‐MI, the study does not extensively explore the pathogenesis of MI itself, which is the primary event associated with CH induced by Tet2 deficiency and its subsequent link to atherosclerosis.^7^, ^8^ In addition, the impact of cGAS/STING inhibition on cardiac fibrosis is not evaluated in our study. Future studies are needed to investigate the broader context of CH‐induced atherosclerosis and its contribution to MI pathogenesis.

## 4 METHODS AND MATERIALS

### 4.1 Mouse models

Animal studies were approved by the Institutional Animal Care Use Committee of the Institute of Biosciences and Technology, Texas A&M University. C57BL/6J LysM‐Cre mice (#004781) and ROSA26‐stop‐EYFP mice (#006148) were obtained from Jackson Laboratories. C57BL/6J Tet2 floxed mice were obtained from Dr. Anjana Rao's laboratory.^19^, ^40^ Mice with myeloid‐restricted Tet2 ablation were generated by crossing Tet2‐floxed mice (Tet2^f/f^) with LysM‐Cre mice and ROSA26‐stop‐EYFP mice. LysM‐Cre mice express Cre in myeloid cells due to a targeted insertion in their endogenous M lysozyme locus. The R26R‐stop‐EYFP mouse is a Cre‐dependent EYFP reporter strain (R26R) produced by targeted insertion of EYFP preceded by a loxP flanked (floxed) transcriptional termination sequence (tpA) into the ROSA26 locus. The R26R allele terminates transcription prematurely, but when crossed with LysMCre mice, the Cre‐mediated excision of the floxed termination sequence in myeloid cells leads to EYFP expression. In addition to the Tet2^f/f^‐LysMCre‐EYFP mouse strain, the 2‐month‐old Tet2^+/+^‐LysMCre‐EYFP mouse strain was used as control group for MI in the same way. Mice tails were cut and boiled in 50 mM NaOH for 1 h and then neutralized in 10 mM Tris–HCl at pH 7.4. PCR was carried out using the EmeraldAmp GT PCR Master Mix (TaKaRa) according to the manual. Genotyping primers are listed in Table S3.

### 4.2 Myocardial infarction surgery

Mice were subjected to permanent ligation of LAD coronary artery to induce MI, using an Olympus SZX7 microscope. Mice were intubated and ventilated with isoflurane supplemented with oxygen (isoflurane 1%–2% vol/vol + 2 L O_2_). The chest wall was shaved, and a thoracotomy was performed in the fourth left intercostal space. The LAD was closed with a 7‐0 monofilament Prolene (Ethicon). Layer‐wise wound closure was performed with a 6‐0 monofilament Prolene (Ethicon). Mice were placed on a temperature‐controlled warming pad TC‐1000 (CWE Inc.) to provide 37°C body temperature. Postoperative pain medication of buprenorphine (.05 mg/kg BW Bupaq, ATC‐Code: QN02AE01, Richter Pharma AG) was given in total for 3 days, three times a day subcutaneously.

### 4.3 Echocardiography

The echocardiographic assessment was conducted before and after MI surgery to evaluate the cardiac function. Briefly, mice were lightly anaesthetized with 1% isoflurane and placed in the supine position on a temperature‐controlled heating platform (VisualSonics mouse table, Fujifilm VisualSonics Inc.) to maintain their body temperature at 37°C. Images were acquired, using the high‐resolution micro‐imaging Vevo 3100 system (VisualSonics Inc.) with a 70‐MHz transducer. 2D and M‐mode images were obtained in the long and short axis views at baseline (Day 0, before surgery) and at 4 weeks post‐MI. EF and other cardiac functions were calculated using Vevo LAB software (Fujifilm VisualSonics Inc.). The summary of echocardiography data was shown in Table S1 and Table S2.

### 4.4 Histological analysis

All mouse hearts were fixed overnight in 10% formalin (Sigma‐Aldrich) then dehydrated with graded ethanol and embedded in paraffin. Sections were cut at 5 μm. Slides were dried at 37°C overnight and then stained with Trichrome Stain (Masson) Kit (HK15, Sigma‐Aldrich) according to the manufacturer's instruction. Stained sections were imaged using an Olympus SZX12 microscope and an Olympus DP21 camera. ImageJ software (National Institute of Health) was used for the measurement and quantification of histological data. The segmental infarct size was calculated as the percentage of infarct area or length in each segment as previously described.^41^

### 4.5 Single‐cell isolation in cardiac tissues

Hearts were collected on Days 0–1, 4, 7 and 16 after MI and perfused ex vivo with PBS. Hearts were weighed and minced in digestion cocktail containing 450 U/mL collagenase I, 125 U/mL collagenase XI, 60 U/mL DNase I and 60 U/mL hyaluronidase (Sigma‐Aldrich) and incubated at 37 °C at 750 rpm for 1 h. Digestion was stopped with 10 mL RPMI 1640 with 10% fetal bovine serum (FBS), and the cells were passed through 40 μm filters to obtain single‐cell suspension. Cells were treated with ACK lysis buffer (Thermo Fisher Scientific) to remove red blood cells.

For sorting of EYFP^+^ cells, the digested suspensions were stained with Live/Dead dye (Biolegend) and sorted on a FACSFusion cell sorter (BD Biosciences). The purity of live EYFP^+^ cells was assessed after sorting, prior to downstream use.

Bone marrow cells were flushed from femurs with ice cold FACS buffer (2% FBS) and passed through 40 μm filters. Peripheral Blood was drawn via cardiac puncture. Red blood cell lysis was removed with ACK lysis buffer, and cells were counted before staining. For calculation of total cell numbers in the heart, normalization to the weight of infarct was performed.

### 4.6 Flow cytometry analysis

Single‐cell suspensions from heart tissues, bone marrow and blood were kept on ice, stained with Live/Dead dye (Biolegend) and blocked with True‐Stain Monocyte Blocker (#426102, Biolegend). Cells were then stained with surface antibodies for 30 min on ice. The following antibodies were used (Table S4): APC‐Ly‐6C, AL‐21 (BD Biosciences), Biotin‐F4/80, A3‐1 (AbD Serotec), Biotin‐CD11c, HL3 (BD Biosciences), Biotin‐I‐A^b^, AF6‐120.1 (BD Biosciences), eFlour450‐CD11b, M1/70 (BD Biosciences), PE‐CD49b, DX5 (BD Biosciences), PE‐CD90, 53‐2.1 (BD Biosciences), PE‐NK1.1, PK136 (BD Biosciences), PE‐Ly‐6G, 1A8 (BD Biosciences), PE‐B220, RA3‐6B2 (BD Biosciences) and Strep‐APC‐CY7 (BD Biosciences) were used to label biotinylated antibodies. Immune cells were identified as previously described.^10^ Monocytes were identified as YFP^hi^ or CD11b^hi^ (CD90/B220/CD49b/NK1.1/Ly‐6G)^lo^ (F4/80/I‐A^b^/CD11c)^lo^ Ly‐6C^hi/lo^. Macrophages/DCs were identified as CD11b^hi^ (CD90/ B220/CD49b/NK1.1/Ly‐6G)^lo^ (F4/80/I‐A^b^/CD11c)^hi^ Ly‐6C^lo^. Neutrophils were identified as YFP^hi^ or CD11b^hi^ (CD90/B220/CD49b/NK1.1/Ly‐6G)^hi^ (F4/80/I‐A^b^/CD11c)^lo^ Ly‐6C^int^.

For FACS staining for γH2AX, dsDNA, cGAS (total), pIRF‐3, pTBK1 and pp65, cells were treated with 4 Fix and Perm buffer (BD Biosciences) according to the manufacturer's instructions. Permeabilized cells were resuspended in BD Perm/Wash buffer (BD Biosciences) and stained with Alexa Fluor 647‐anti‐γH2AX (JBW301 Millipore Sigma), dsDNA (HYB331‐01 Santa Cruz Biotechnology), Alexa Fluor 647‐pIRF‐3 (Ser396) (D6O1M Cell Signaling Technology), Alexa Fluor 555‐pTBK1 (Ser172) (D52C2 Cell Signaling Technology), Alexa Fluor 594‐pp65 (Ser536) (93H1 Cell Signaling Technology) antibody or BUV395 Rabbit Anti‐Active Caspase‐3 antibody (BD Biosciences, Cat. 570181) for 30 min.

For staining of total or cytosolic cGAS, cells were permeabilized using Foxp3/Transcription Factor Staining Buffer Set (eBioscience, Cat# 00‐5523) according to the manufacturer's instructions. Cells were stained with Alexa Fluor 647 anti‐cGAS (E5V3W, Cell Signaling Technology) 1 h on the ice. For the apoptosis assay, cells were stained using the APC Annexin V Apoptosis Detection Kit with 7‐AAD (Biolegend, Cat. 640930) according to the manufacturer's instructions. All data were collected using an LSRII (BD Biosciences) flow cytometer and analysed using FlowJo (TreeStar Inc.).

### 4.7 Immunofluorescence staining

For immunofluorescence staining for γH2AX, dsDNA, cGAS, R‐loop and G4, EYFP^+^ cells were purified by cell sorting as described above and concentrated into 8 Well Chambered Cover Glass with #1.5 high‐performance cover glass (#C8‐1.5H‐N, Cellvis). Cells were fixed with freshly prepared 4% paraformaldehyde solution for 15 min at room temperature. After three times wash with PBS, cells were permeabilized with .2% Triton X‐100 in PBS, followed by blocking with 1% BSA/.05% Tween‐20/PBS at room temperature for 1 h. Then cells were incubated with primary antibodies against phosphohistone H2AX (γ‐H2AX, Ser‐139 Millipore Sigma, #05636), dsDNA (HYB331‐01 Santa Cruz Biotechnology), cGAS Rabbit mAb (#15102 Cell Signaling Technology), Anti‐DNA‐RNA Hybrid Antibody, clone S9.6 (MABE1095 Millipore Sigma) or BG4‐Ig antibody (MABE917 Millipore Sigma) in the blocking buffer overnight at 4°C. Cells were then washed five times with PBS and incubated with secondary antibodies goat anti‐mouse/rabbit IgG conjugated with Alexa Fluor 555 or 647 (Table S4) in the blocking buffer at room temperature for 1 h. Cells were then stained with DAPI for further imaging. W1 Yokogawa Ti2 Nikon Spinning Disk Confocal Microscope was used for imaging acquisition and analysis. The captured images were analysed by the Nikon Elements imaging processing software (Nikon, NIS‐element AR version 4.0) or the ImageJ programme (NIH).

### 4.8 R‐loop and G‐quadruplex detection

For flow cytometry‐based detection of R‐loops and G4s, cells were fixed with 4% paraformaldehyde in PBS for 12 min at 25°C, followed by permeabilized at 4°C overnight by using the intracellular transcription factor staining kit (Invitrogen). Cells were then washed and incubated with 1:50 dilutions of Anti‐DNA‐RNA Hybrid Antibody, clone S9.6 (MABE1095 Millipore Sigma) for 30 min at 4°C followed by washing, subsequent staining with anti‐mouse secondary antibody (1:1000, #A‐32727, Invitrogen). For RNASE H digestion and R‐loop quantification, cells were fixed, permeabilized and treated with 20 units of RNASE H (NEB #M0297S) in 100 μL of digestion buffer diluted in water for 2 h at 37°C before proceeding to R‐loops staining. For G4 quantification, cells were treated with Ambion RNASE A (1:50, AM2269; Invitrogen) for 30 min at 25°C followed by washing and incubation with 1:100 dilutions of BG4‐Ig antibody (MABE917 Millipore Sigma) for 30 min at 25 or 4°C. This was followed by washing and subsequent staining with anti‐mouse IgG1 fluorophore‐conjugated antibody (1:1000, #A21236, Invitrogen).

### 4.9 RNA isolation and real‐time quantitative PCR (RT‐qPCR)

Total RNA from sorted EYFP^+^ cells was extracted using TRIzol reagent (Invitrogen) by following the manufacturer's instructions. DNA was removed through DNase I digestion. cDNA synthesis was achieved using a PrimeScript 1st strand cDNA Synthesis Kit (Takara). Gene expression was quantified on ViiA 7 Real‐Time PCR System (Applied Biosystems) using 2× Universal SYBR Green Fast qPCR Mix (ABclonal). The data were presented as accumulation index (2^△△Ct^). All the primers were synthesized from Integrated DNA Technologies and listed in Table S5.

### 4.10 In vivo inhibition of the STING

The STING inhibitor, H‐151, was purchased from InvivoGen (inh‐h151). H‐151 was dissolved in sterilized DMSO to make a 10 mg/mL stock solution. The stock solution was then diluted with 10% Tween 80 in PBS, with the final concentration of DMSO in the working solution at 0.002%. Mice were injected intraperitoneally either with 750 nmol H‐151 in 200 μL PBS or an equivalent volume of 0.002% DMSO 10% Tween‐80 in PBS (as solvent control) daily for 4 days based on a previous report.^33^ The first injection was performed 4 h before the MI surgery.

### 4.11 2′,3′‐Cyclic GAMP ELISA quantification

To quantify 2′3′‐cGAMP levels in the serum and EYFP^+^ cells, 2 × 106 cells were thoroughly resuspended in 100 μL M‐PER Mammalian Protein Extraction Reagent (#78503, Thermo Scientific). Lysates were incubated on ice for 30 min, centrifuged at 16 000 × *g*, 4°C for 10 min, and 2′3′‐cGAMP levels were quantified using the 2′3′cGAMP ELISA Kit (Arbor Assays) according to the manufacturer's instructions.

### 4.12 Cytokine discovery assay

Plasma samples were collected at Day 1 post‐MI with DMSO or H‐151 treatment. Cytokines and chemokines were detected by using a Mouse Cytokine/Chemokine 44‐Plex Discovery Assay Array (MD44) provided by Eve Technologies. The 44‐plex Discovery assay is based on the Luminex technology and utilizes the Millipore assay that contains fluorescent color‐coded beads pre‐coated with capture antibodies targeting 44 specific cytokines. Fluorescence intensity values derived from the assays were reported. The absolute protein concentrations were calculated from the standard curve and reported as pg/mL.

## AUTHOR CONTRIBUTIONS

Yun Huang, James F. Martin and Yubin Zhou directed and oversaw the project. Yaling Dou performed most animal‐related work, immunofluorescent and flow cytometry analysis. Yan Zhang and Thuy Tien Tran performed myocardium infarction procedure. Logan Rivera supported the statistical analysis. Tingting Hong and Shaohai Fang provided essential resources and key intellectual inputs to support this study. All the authors participated in the discussion, data interpretation and manuscript preparation or discussion.

## CONFLICT OF INTEREST STATEMENT

JFM is a cofounder and owns shares in YAP Therapetucis.

## ETHICS STATEMENT

All animal procedures were approved by the Institutional Animal Care and Use Committee of the Institute of Biosciences and Technology, Texas A&M University. The experiments strictly adhered to the tumor induction guidelines for mice.

## CONSENT FOR PUBLICATION

All authors have reviewed and agreed on the contents of the manuscript.

## Supporting information

Supporting Information

Supporting Information

Supporting Information

Supporting Information

Supporting Information

Supporting Information

Supporting Information

## Data Availability

All data generated or analysed during this study are included in this published article and its supplementary information files.
