# Supplementary material for: Inhibiting the cGAS‐STING pathway in myeloid cells effectively improves myocardial healing related to TET2 deficiency‐induced DNA damage response
Source: Clin Transl Med. 2024 Jun 22;14(6):e1741. doi: 10.1002/ctm2.1741 (PMC11193135; doi:10.1002/ctm2.1741)
Supplement: Supplementary file 1 — Supporting Information [file CTM2-14-e1741-s004.docx]

**Supplementary information**

**Inhibiting the cGAS-STING pathway in myeloid cells effectively improves myocardial healing related to TET2 deficiency-induced DNA damage response**

Yaling Dou^1^, Yan Zhang^1^, Logan Rivera^1^, Tingting Hong^1^, Shaohai Fang^1^, Thuy Tien Tran^2^, Yubin Zhou^1^, James F. Martin^2#^, Yun Huang^1,3#^

1. Institute of Biosciences and Technology, Texas A&M University, Houston, TX 77030, USA
2. Department of Integrative Physiology, Baylor College of Medicine, and Texas Heart Institute Houston, TX 77030, USA.
3. Department of Translational Medical Sciences, School of Medicine, Texas A&M University, Houston, TX 77030, USA

# Correspondence: jfmartin@bcm.edu; yun.huang@tamu.edu (lead contact)

**Figure S1.** **Myeloid-specific Tet2 depletion promotes neutrophil expansion in mice following LAD ligation-induced MI. (Related to Figure 1)**

(A) Representative genotyping results indicative of the Tet2 flox, LysMCre (L) and EYFP (Y) status.

(B) Two gating strategies for identifying Ly6G+ neutrophils, monocytes (Ly6C-high and Ly6C-low), and macrophages / dendritic cells (DCs). Top, the traditional gating strategy based on a previous publication using CD11b; Bottom, the gating strategy within EYFP+ myeloid cells.

(C) Representative immunofluorescence images (left) and quantification (right) of the MPO+ neutrophils in the myocardial infarct areas of the control and Tet2KO mice at day 1 post-MI (n = 6-8 scar areas). Green, MPO; Blue, DAPI; Red, WGA. Data were shown as mean ± SD (**** p < 0.0001, two-sided unpaired Student’s t-test).

(D-E) Quantification of the population of Ly6G+ neutrophils, monocytes (Ly6C-high and Ly6C-low), and macrophages / dendritic cells (DCs) in the peripheral blood (D) and bone marrow (E) from both the control and Tet2-KO groups at 0, 1, 4, 7, or 16 days after MI (n = 4). Data were shown as mean ± SD (* p < 0.05; two-sided unpaired Student’s t-test).

**Figure S2. Tet2-deficient myeloid cells exhibit increased DNA damage. (Related to Figure 2)**

(A) Assessment of neutrophil viability following MI. Shown were representative flow cytometry profiles reporting the levels of active Caspase-3 and Annexin-V/7-AAD staining in EFYP+ cells and Ly6G+ neutrophils isolated from the control and Tet2-KO mice at day 1 after MI.

(B) Quantification of the mean fluorescence intensity (MFI) of the γH2AX levels for the indicated groups (n = 3). Data were shown as mean ± SD (* p < 0.05; two-sided unpaired Student’s t-test).

(C) Representative immunofluorescence images of EYFP+ cells purified from the bone marrow of control (Tet2^+/+^) and Tet2-KO (Tet2^f/f^) mice at Day 0, 1, 4, 7, 16 following LAD ligation procedures. Scale bar, 5 µm.

(D) Representative immunofluorescence images (left) and quantification (right) of the double stranded DNA (dsDNA) levels in EYFP+ cells obtained from the myocardial infarct areas of the control or Tet2KO mice at Day 1 post-MI (n = 51 cells). Scale bar: 5 µm. Data were shown as mean ± SD (**** p < 0.0001, two-sided unpaired Student’s t-test).

**Figure S3. Tet2 deficiency causes augmented activation of the cGAS-STING pathway. (Related to Figure 3)**

Data were shown as mean ± SD (** p < 0.005, * p < 0.05; two-sided unpaired Student’s t-test).

(A) Flow cytometry analysis on the expression levels of cGAS in EFYP+ cells obtained from the bone marrow, peripheral blood and myocardial infarct areas of the control and Tet2KO mice at Day 1 after MI.

(B) Representative immunofluorescence images (left) and quantification (right) of the cGAS protein levels in EYFP+ cells obtained from the infarcted areas at Day 1 post-MI (n = 30 cells). Scale bar, 20 µm.

(C) Immunoblot analysis on phosphorylated TBK1 (p-TKB1) and p65 (p-p65) in EFYP+ cells obtained from myocardial infarct areas in the control and Tet2KO mice at day 1 after MI. Total TBK1, p65 and GAPDH were used as controls.

(D) The statistical quantification of the mean fluorescence intensity (MFI) measured by flow cytometry, indicating the levels of phosphorylated TBK1, IRF3 and p65 in EFYP+ cells purified from bone marrow and peripheral blood of the control and Tet2KO mice at Day 0, 1, 4, 7 after MI (n = 3).

(E) Realtime qPCR analysis of *Il6* and *Il1β* expression in EFYP+ cells obtained from myocardial infarct areas in the control and Tet2KO mice at day 1 after MI (n = 3). Data were shown as mean ± SD; ns, not significant (two-sided unpaired Student’s t-test).
