# Supplementary material for: Inhibiting the cGAS‐STING pathway in myeloid cells effectively improves myocardial healing related to TET2 deficiency‐induced DNA damage response
Source: Clin Transl Med. 2024 Jun 22;14(6):e1741. doi: 10.1002/ctm2.1741 (PMC11193135; doi:10.1002/ctm2.1741)
Supplement: Supplementary file 2 — Supporting Information [file CTM2-14-e1741-s001.docx]

**Table S1. Summary of Echo data**
