# Supplementary material for: Inhibiting the cGAS‐STING pathway in myeloid cells effectively improves myocardial healing related to TET2 deficiency‐induced DNA damage response
Source: Clin Transl Med. 2024 Jun 22;14(6):e1741. doi: 10.1002/ctm2.1741 (PMC11193135; doi:10.1002/ctm2.1741)
Supplement: Supplementary file 7 — Supporting Information [file CTM2-14-e1741-s007.pptx]

## Slide 1
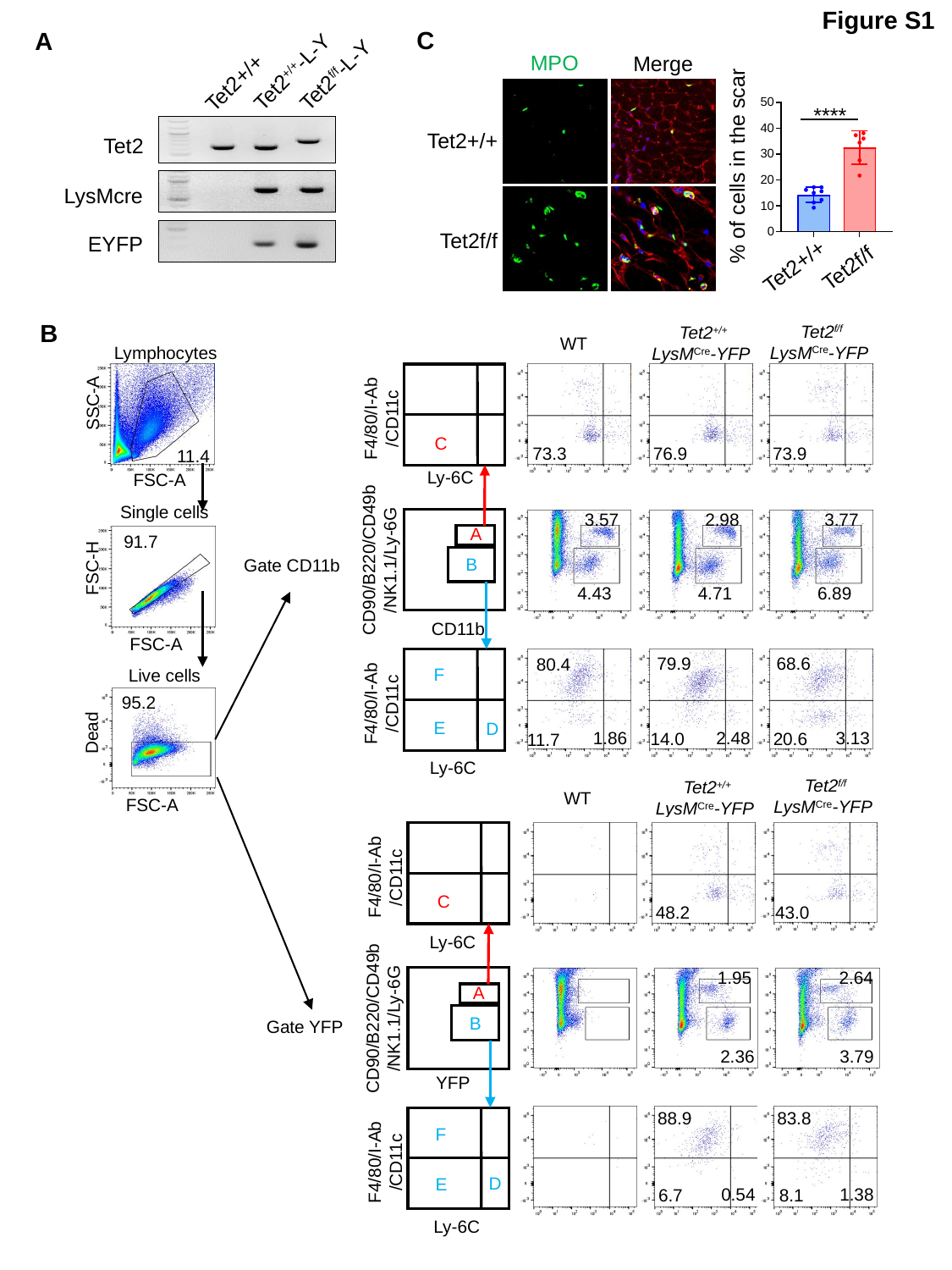

Figure S1
Tet2+/+-L-Y
Tet2f/f-L-Y
Tet2+/+
Tet2
LysMcre
EYFP
C
A
MPO
Merge
****
Tet2+/+
% of cells in the scar
Tet2f/f
Tet2+/+
Tet2f/f
B
Tet2f/f
LysMCre-YFP
Tet2+/+
LysMCre-YFP
WT
Lymphocytes
SSC-A
F4/80/I-Ab
/CD11c
C
76.9
73.9
73.3
11.4
Ly-6C
FSC-A
Single cells
3.77
2.98
3.57
A
91.7
CD90/B220/CD49b
/NK1.1/Ly-6G
B
Gate CD11b
FSC-H
6.89
4.71
4.43
CD11b
FSC-A
79.9
68.6
80.4
F
Live cells
F4/80/I-Ab
/CD11c
95.2
Dead
E
D
2.48
3.13
1.86
14.0
20.6
11.7
Ly-6C
Tet2f/f
LysMCre-YFP
Tet2+/+
LysMCre-YFP
WT
FSC-A
F4/80/I-Ab
/CD11c
C
43.0
48.2
Ly-6C
2.64
1.95
A
CD90/B220/CD49b
/NK1.1/Ly-6G
B
Gate YFP
2.36
3.79
YFP
88.9
83.8
F
F4/80/I-Ab
/CD11c
D
E
0.54
1.38
8.1
6.7
Ly-6C

## Slide 2
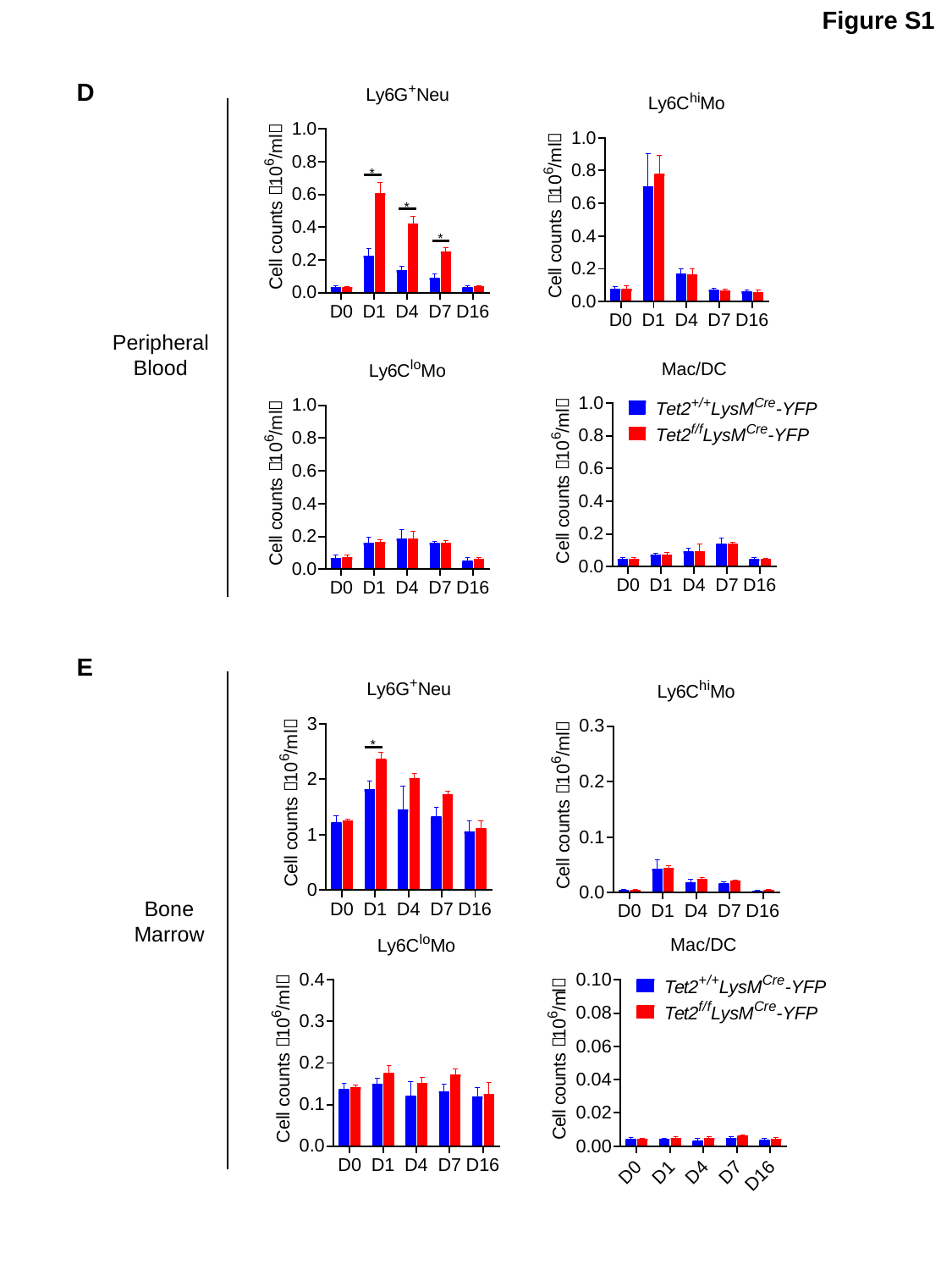

Figure S1
D
Peripheral
Blood
E
Bone Marrow

## Slide 3
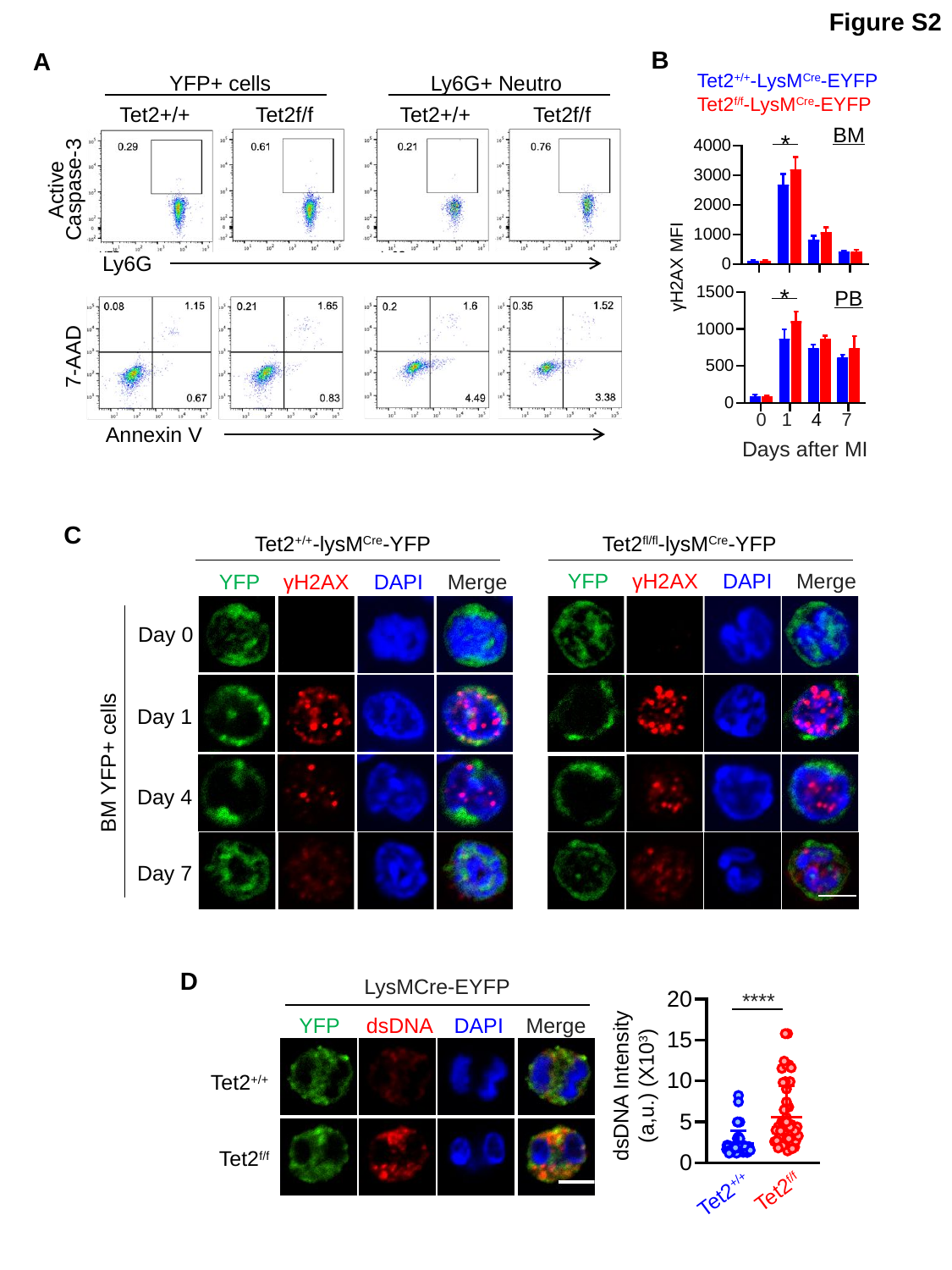

Figure S2
B
A
Tet2+/+-LysMCre-EYFP
Tet2f/f-LysMCre-EYFP
YFP+ cells
Ly6G+ Neutro
Tet2+/+
Tet2f/f
Tet2+/+
Tet2f/f
Active Caspase-3
Ly6G
7-AAD
Annexin V
BM
*
γH2AX MFI
*
PB
0
1
4
7
Days after MI
C
Tet2+/+-lysMCre-YFP
Tet2fl/fl-lysMCre-YFP
 YFP
γH2AX
DAPI
Merge
 YFP
γH2AX
DAPI
Merge
Day 0
Day 1
BM YFP+ cells
Day 4
Day 7
****
 dsDNA Intensity
(a,u.) (X103)
Tet2f/f
Tet2+/+
D
LysMCre-EYFP
 YFP
dsDNA
DAPI
Merge
Tet2+/+
Tet2f/f

## Slide 4
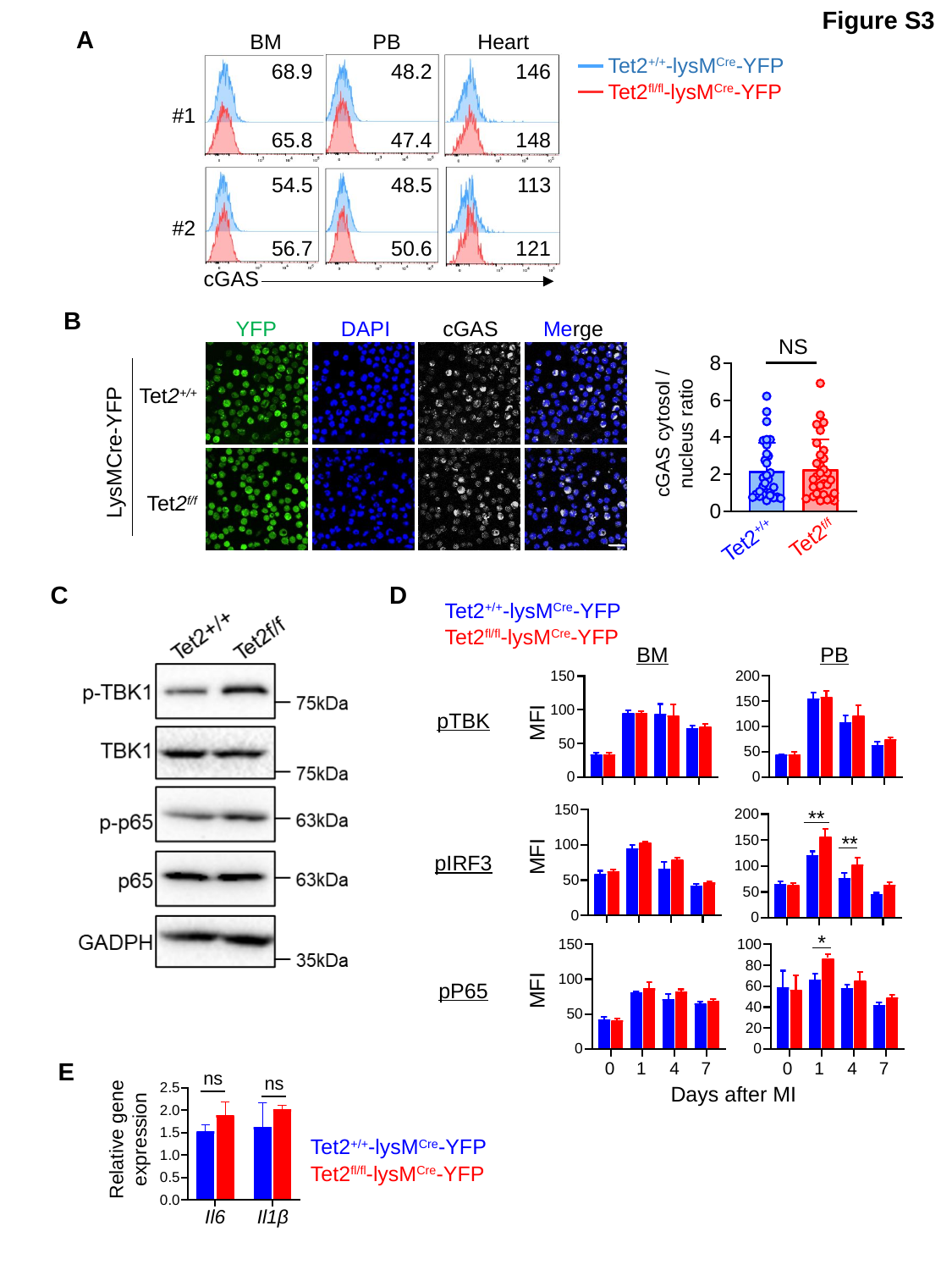

Figure S3
A
BM
PB
Heart
Tet2+/+-lysMCre-YFP
Tet2fl/fl-lysMCre-YFP
68.9
48.2
146
#1
65.8
47.4
148
54.5
48.5
113
#2
56.7
50.6
121
cGAS
B
YFP
DAPI
cGAS
Merge
NS
cGAS cytosol / nucleus ratio
Tet2f/f
Tet2+/+
Tet2+/+
LysMCre-YFP
Tet2f/f
C
D
Tet2+/+-lysMCre-YFP
Tet2fl/fl-lysMCre-YFP
BM
PB
pTBK
MFI
**
**
MFI
pIRF3
*
pP65
MFI
0
1
4
7
0
1
4
7
Days after MI
E
ns
ns
Relative gene expression
Tet2+/+-lysMCre-YFP
Tet2fl/fl-lysMCre-YFP
Il6
Il1β
