# Supplementary material for: Inhibiting the cGAS‐STING pathway in myeloid cells effectively improves myocardial healing related to TET2 deficiency‐induced DNA damage response
Source: Clin Transl Med. 2024 Jun 22;14(6):e1741. doi: 10.1002/ctm2.1741 (PMC11193135; doi:10.1002/ctm2.1741)
Supplement: Supplementary file 6 [file CTM2-14-e1741-s005.docx]

Supplementary Table 5. RT-PCR primer sequences.

| Gene | Direction | 5’ → 3’ |
|---|---|---|
| *Mouse Gapdh* | Forward | GACTTCAACAGCAACTCCCAC |
| | Reverse | TCCACCACCCTGTTGCTGTA |
| *Mouse Ifit1* | Forward | CAAGGCAGGTTTCTGAGGAG |
| | Reverse | GACCTGGTCACCATCAGCAT |
| *Mouse Ifit3* | Forward | TTCCCAGCAGCACAGAAAC |
| | Reverse | AAATTCCAGGTGAAATGGCA |
| *Mouse Ifil44* | Forward | CTGATTACAAAAGAAGACATGACAGAC |
| | Reverse | AGGCAAAACCAAAGACTCCA |
| *Mouse Isg15* | Forward | CTAGAGCTAGAGCCTGCAG |
| | Reverse | AGTTAGTCACGGACACCAG |
| *Mouse Irf7* | Forward | CAATTCAGGGGATCCAGTTG |
| | Reverse | AGCATTGCTGAGGCTCACTT |
| *Mouse Cxcl10* | Forward | CCAAGTGCTGCCGTCATTTTC |
| | Reverse | GGCTCGCAGGGATGATTTCAA |
