# Supplementary material for: Inhibiting the cGAS‐STING pathway in myeloid cells effectively improves myocardial healing related to TET2 deficiency‐induced DNA damage response
Source: Clin Transl Med. 2024 Jun 22;14(6):e1741. doi: 10.1002/ctm2.1741 (PMC11193135; doi:10.1002/ctm2.1741)
Supplement: Supplementary file 7 [file CTM2-14-e1741-s006.docx]

Supplementary Table 3: Mice genotyping primer sequences.

Genotype	Primer Type	5’ → 3’	Size (bp)
Tet2 flox	Forward	GCCCAAGAAAGCCAAGACCAAGAA	WT = 650, FL =780
	Reverse	AAGGAGGGGACTTTTACCTCTCAGAGCAA	
Rosa2-EYFP	Forward (Wildtype)	GGAGCGGGAGAAATGGATATG	WT = 600, Mut = 320
	Forward (Mutant)	AAGACCGCGAAGAGTTTGTC	
	Reverse (Common)	AAAGTCGCTCTGAGTTGTTAT	
LysMCre	Forward (Wildtype)	TTACAGTCGGCCAGGCTGAC	WT = 350, Mut = ~700
	Forward (Mutant)	CCCAGAAATGCCAGATTACG	
	Reverse (Common)	CTTGGGCTGCCAGAATTTCTC	
